# Measuring the sustainability of beef supply chain with rapid appraisal for beef supply chain

**DOI:** 10.14202/vetworld.2021.2488-2507

**Published:** 2021-09-23

**Authors:** Aries Susanty, Ratna Purwaningsih, Haryo Santoso, Anggun Novi Arista, Benny Tjahjono

**Affiliations:** 1 Department of Industrial Engineering, Faculty of Engineering, Diponegoro University, Tembalang, Semarang, Central Java, 50275, Indonesia; 2 Centre for Business in Society, Coventry University, Coventry, United Kingdom.

**Keywords:** beef supply chain, indicators, rapid appraisal for beef supply chain, sustainability

## Abstract

**Background and Aim:**

Nationally, there has always been a gap between the demand for beef and its supply, although supply growth is proportional with demand growth and even exceeds it in some regions in Indonesia. This research study aims to measure the sustainability status of the beef supply chain and applies the developed measurement system to a specific beef supply chain by identifying suitable indicators and their scale. Moreover, this research study provides some recommendations for the improvement of the sustainability status of the beef supply chain.

**Materials and Methods:**

In this research study, 11 and nine indicators were analyzed to assess the sustainability status of the beef supply chain at the farm and slaughterhouse chain levels. A rapid appraisal for beef supply chain was applied to rapidly assess the sustainability status of beef supply chains using Multidimensional Scaling (MDS). The Delphi method was utilized as an iterative process to collect data and obtain consensus of experts’ judgments regarding the policies that should be implemented to improve the most sensitive indicator affecting the economic, social, and environmental dimensions.

**Results:**

Analysis of ordination with MDS shows the regional sustainability index value for multidimensional approaches of beef cattle farms and beef slaughterhouses. The sustainability index value for beef cattle farms was 56.14 (moderately sustainable), 48.02 (fairly unsustainable), and 48.77 (fairly unsustainable) in Semarang, Sragen, and Boyolali, respectively. Moreover, the sustainability index value for beef slaughterhouses was 47.05 (fairly unsustainable), 54.83 (moderately sustainable), and 54.19 (moderately sustainable) in Semarang, Sragen, and Boyolali, respectively. Policy recommendation was focused on the basis of the results of leverage analysis, which highlighted the most indicative factor affecting sustainability for each dimension.

**Conclusion:**

Measurement results revealed that the achievement of beef supply chain sustainability requires targeted efforts through the deployment of several policies as the current status of sustainability in beef farms and beef slaughterhouses was only inclined toward moderately sustainable and fairly unsustainable. Although all the surveyed regions in this study can meet the regional needs of beef meat on their own and even distribute the excess to other regions, none of the beef supply chains of the surveyed region indicated good sustainability.

## Introduction

In Indonesia, the average beef meat consumption increased from 1.44 kg/capita/year in 2006 to 1.90 kg/capita/year in 2019. It was forecasted to amount to about 2.12 kg/capita/year in 2025 [1]. Several factors contributed to the growth of Indonesian demand for beef meat, increasing middle-class income, growing population, increasing urbanization, and shifting away from a pattern of consumption from staple foods toward high-value agricultural products [2-6]. As of date, domestic production can only supply about 45% of Indonesian demand for beef meat [7]. Nationally, there has always been a gap between the demand for beef meat and its supply, although supply growth is proportional with demand growth and even exceeds it sometimes in some places (such as in Central Java, East Java, South Sulawesi, and East Nusa Tenggara). The Indonesian government has tried to overcome this situation by developing a beef meat self-sufficiency program since 2000 (Program Swasembada Daging Sapi, PSDS 2001, 2005, and 2014). However, this program lacked effectiveness and was deemed unsuccessful; the gap between supply and demand on beef remains and may tend to increase [8].

Permani [5] revealed several factors that make Indonesian beef meat supply fall short of domestic demand. These factors included poor management practices, limited education of farmers, minor benefit to farmers, high prices of beef cattle feed, scarcity of forage during the dry season, limited access to high-quality genetics, limited access to bank loans, and the conversion of agricultural land for housing, businesses, and industry, which may affect the quality of pasture and feed resources. Poor management practice and limited education are two factors originating from a situation where smallholder farming dominates meat production in Indonesia. In this case, the smallholder farming system constitutes approximately 90% of meat production, with about 6.5 million farmers living in rural areas. The commercial farmers and large beef cattle companies constitute 1% and 10% of meat production, respectively [9-11]. The small farmers only have three to four cows on average, and most of them have little or even no prior experience in livestock education [6,12,13]. Then, there is the minor benefit to farmers related to the absence of government regulation to protect farmers from middlemen. Although the cattle prices in the market are high, the farmers cannot benefit from this because of their poor bargaining position [14].

The growth of demand and lack of supply raises important questions around how the beef meat supply will meet this demand efficiently and sustainably [15]. In this case, there is a growing need for sustainability in beef supply chains to decrease the impact on the environment when a supply chain’s economic and social needs are sought to be achieved. In other words, to get optimum benefit, the development of the supply side needs to meet the criteria of sustainable development associated with economic, social, and environmental interests [16]. For example, according to Pashaei *et al*. [17], the economic issue in the beef supply chain is related to the profitability of the farm as well as the impact on the local economy. The social issues are related to labor rights and food safety, whereas environmental issues related to climate change, water and energy use, water pollution, soil degradation, land use change, and biodiversity loss. In addition to Pashaei *et al*. [17], economic, social, and environmental issues in the beef supply chain are also discussed by several authors [18-21]. For the beef supply chain, meeting environmental issue criteria are also an important factor because the growth of meat production will lead to the use of a large amount of water and energy and may release a large quantity of waste and gaseous emissions into the environment [22]. In all the meat processing chain steps, water is essential because the equipment, machines, and processing areas in the meat industry are designated to work in humid conditions requiring wet cleaning. Those conditions affect the consumption of water and the discharge of unused water polluted with raw materials, products, and cleaning chemicals [23]. Then, energy is also used throughout the meat chain. It is used to control the temperature regime, that is, heat treatments such as cooking, boiling, sterilizing, drying, pasteurizing, and smoking and cooling (mainly chilling and freezing) [23]. Moreover, energy is used for transportation purposes [24]. In relation to wastes, beef meat supply produces solid waste as well as wastewater. There are two main types of solid waste. First, there are inedible products, such as fat, bones, legs, head, hair, skins, and offal. Second, there are packaging materials, such as plastic, paper, and metal. The wastewater has resulted from several activities, such as the hygienic disposal of carcasses and offal, washing of livestock, clean working environments and equipment, personal hygiene of workers, and efficient truck washing [25]. It is important to note that wastewater usually contains some pollutants such as fat, blood, manure, meat, meat extracts, undigested stomach contents, dirt, and cleaning agents. Hence, the importance of wastewater indicators is the amount of wastewater discharged and the pollutant load that is generated. Both are determined by the type of meat and meat products being manufactured and by the technological environment.

Assessing sustainability is a complex process and needs the participation and collaboration of all actors in the system to answer such essential questions [26]. What are the relationships between the various indicators of sustainability? What indicators should and can be measured, and how are the results interpreted so that farmers can improve their productivity? What kinds of practices improve sustainability? Which channels can best facilitate the dissemination and adoption of practices in different conditions? Hence, in light of those questions and as part of a study for assessing sustainability in the beef supply chain, this study has several research objectives.

• First, identify sustainability indicators in economic, environmental, and social dimensions that are best suited to assess the sustainability of beef supply chains.
• Second, identify the current sustainability level of beef supply chains in Central Java Province, which is measured by the Rapid Appraisal for Beef Supply Chain (RAPBEEF) method.
• Third, identify the most suitable improvement that should be carried out to increase the sustainability level of each dimension.

## Materials and Methods

### Ethical approval

This study does not require ethical approval.

### Beef supply chain in Indonesia

In the past 16 years (from 2000 to 2016), beef cattle in Indonesia have tended to increase. In 2000, the beef cattle population was 11,008,017 heads; 10 years later, in 2010, the beef cattle population increased to 13,581,570 heads (about 2.34%/year). Six years later, in 2016, the population of beef cattle increased to 16,092,561 heads (about 3.08%/year). In 2016, the largest population of beef cattle was located in East Java (28.18%), followed by Central Java (10.45%), South Sulawesi (8.41%), West Nusa Tenggara (6.84%), and East Nusa Tenggara (5.79%) [27,28].

**Figure-1:**
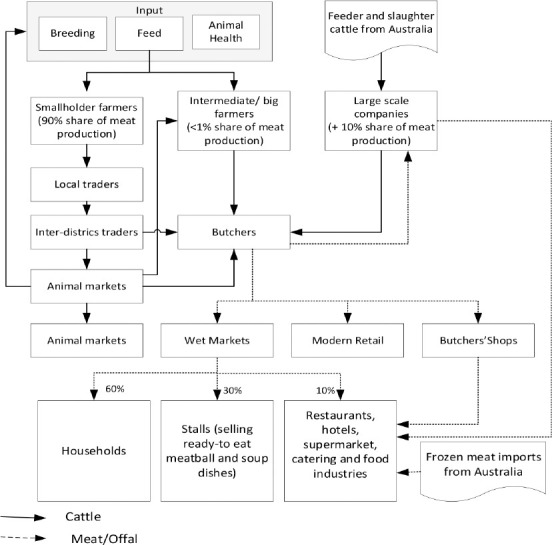
The structure of the Indonesian beef cattle supplies chain.

The structure of the Indonesian beef cattle supply chain is shown in Figure-1 [14,29]. The source of beef cattle production can be differentiated into three, smallholder farmers, intermediate or big farmers, and large-scale companies [14]. The smallholder farmers and intermediate or big farmers receive input from breeding, feed, and animal health. There are three approaches of breeding systems in Indonesia: Natural breeding, where households use their bulls; natural breeding using group bulls; and artificial insemination (AI). The feed inputs for cattle production in Indonesia are varied. It depends on the region of the farming system. The feed inputs range from the cut-and-carry of crop residues and grasses (e.g., in Java); open grazing of cattle in the grass and Shrubland in Eastern Indonesia; tree forages in intensive and extensive systems; and residues from plantation crops in Sumatra and Kalimantan. Then, in relation to animal health, veterinary services are provided by the Animal Health Division that forms a separate line agency within the Directorate General of Livestock and Animal Health Services down to local levels. The division oversees animal health centers (“Pusat Kesehatan Hewan/Puskeswan”) down to subdistrict levels staffed by veterinarians or lower-level “animal paramedics” [29].

Most of the smallholder farmers sell their cattle through the local market, and the price of cattle is calculated on the basis of live weight estimation by the farmer and local traders. Then, local traders sell the cattle to inter-district traders, and inter-district traders sell the cattle to the animal market. In this case, besides selling their cattle to a local trader, the smallholder farmer can also sell their cattle directly to animal markets. During Id-ul Adha (one of the Muslim holidays), many cattle are sold directly to consumers. Farmers seldom sell cattle themselves at a market or to butchers but rather sell through collectors/brokers or local traders. The cattle can then change hands several times to be aggregated in larger lots (for inter-island traders or feedlots) or regular buyers (especially butchers). Unlike smallholder farmers, most feedlots and intermediate farmers sell their cattle directly to buyers or butchers. The prices are determined on the basis of live weight measured using weighing scales. Local butchers slaughter cattle in slaughterhouses that are owned by the government [14,29].

Local butchers usually sell the meat to consumers. Indonesia’s end consumers of beef buy meat in three retail formats: Wet markets, modern retail, and butchers’ shops [30]. In wet markets, 60% of customers are households, followed by stalls selling ready-to-eat meatball and soup dishes constituting as much as 30%, and then by restaurants and supermarkets to the extent of 10% [31]. The demand for meat from large restaurants, hotels, and catering and food industries is met by local production from butchers and feedlots (65%) and frozen meat imports (35%) from Australia [14]. In this case, imported beef is sold in modern retail (mainly at supermarkets and hypermarkets, approximately 62% of imported beef) and is used in the foodservice sector [3,32]. Besides modern retail, approximately 20% of imported beef is sold in the wet market, some 17% is sold in butcheries, and then some 2% is sold in online retailers [32]. Indonesia imports live animals and frozen meat for approximately 30% of its national consumption [33,34], primarily from New Zealand and Australia [3,34]; these countries had a market share of 15% and 81% in 2016, respectively [3]. Most imports from Australia are livestock for slaughter, but others are for breeding and fattening [20]. In 2018, Indonesia’s sources of imported meat, both fresh and frozen, were India for about 49%, Australia for 42%, the United States and New Zealand for 3% each, as well as other countries such as Spain, Canada, and Singapore [35].

### The concept of sustainability chain management and the measurement

World Commission on Environment and Development stated that sustainability is related to the fulfillment of the three pillars of sustainable development, or the so-called Triple-Bottom-Line (3BL), which stresses economic, social, and environmental performance to enhance human beings’ quality of life. Similarly, the World Business Council of Sustainable Development noted that sustainability refers to the synchronized pursuit of economic prosperity, social equity, and environmental quality, where enterprises targeting sustainability should perform not against a single financial bottom line but the 3BL [36]. Moreover, Elkington [37,38] emphasized the concept of sustainability as the intersection of economic, social, and environmental components.

At present, the intersection between sustainability and supply chain management is one of the most promising areas within the supply chain literature, having become a relevant topic for researchers and professionals [39]. It focuses on the integration of three sustainability dimensions from Elkington [37,38] (economic, social, and environmental) into the concept of supply chain management [40]. The intersection between two concepts is known as sustainable supply chain management (SSCM). Then, referring to Carter and Roger [41], SSCM can be defined as a strategic and transparent integration of economic, social, and environmental aspects in systemic coordination of organizational processes to improve enterprises’ economic performance in the long run. Seuring and Müller [42] propose the definition of SSCM as the management of material, information, and capital flow, as well as cooperation among enterprises along the supply chain, while considering goals from all three dimensions of sustainable development, that is, economic, social, and environmental, which are derived from requirements of customers and stakeholders. Besides Carter and Roger [41], as well as Seuring and Müller [42], more than 16 different definitions exist for SSCM [43] because of the lack of agreement on the meaning of SSCM [44].

Not only the lack of agreement on the meaning of SSCM but also the lack of agreement related to indicators should be used to measure sustainability. In this case, the sustainability measurement can be seen as “a tool that can support decision-makers to select what activities they should do and should not do in an attempt to make society more sustainable” [45] or as a tool to guarantee that strategies and activities make an optimal impact on sustainable development by referring to the dimensions of sustainability [46]. Recently, measurement of sustainability through indicators has been widely employed by the scientific community, governments, and policymakers. The indicators can serve as a great instrument in assessing economic, social, and environmental issues. The selection of indicators is often subjective, and the choice of an indicator is determined by several factors, such as measurability, cost-effectiveness, ease of understanding, scientific reliability, and international comparability [47]. Then, according to the 15 authors, several indicators used to measure sustainability in general, or sustainability in the food or beef supply chain, are shown in Table-1 [16-21,48-56].

**Table-1:** Indicators for measuring sustainability in general food or beef supply chain.

Economic		Social		Environmental	
		
Indicators	Reference	Indicators	Reference	Indicators	Reference
Profitability	[17-19,48,49]	Food safety	[17-19,48, 50]	Energy used	[17,19-21,48-55]
Income distribution	[16,19,49,50]	Human health and safety	[20,50-52,55]	Global warming–GHG emissions	[19,48,49,52,53,55]
Investment cost (Material cost, energy cost, cost saved, operational and capital cost; insurance cost; Research and Development cost)	[20,21,50,51]	Number of employees owned by the enterprises	[21,49,50,52,54]	Water consumption	[21,50,52,54-56]
Economic growth (local/national)	[17,19,20]	Working condition	[17,48,51,56]	Land used	[19,48,49,55,56]
Rate of employee give some suggestions about quality social, environmental, health and safety	[51-53]	Gender equality	[19,21,54,56]	Waste disposal	[18,49,54,56]
Volatility (price variation)	[17,48]	Animal welfare	[19,48,56]	Material used	[17,18,49,51,52]
Self-sufficiency	[18,54]	Number of employees trained/to be trained	[49,51,53]	Waste utilization	[16,50,51]
Cost associated with non-compliance	[51,52]	Employability	[18,48]	Atmosphere	[20,48]
Employment	[19]	Food quality	[19,48]	Water quality	[17,20]
Place to sell beef cattle	[16]	Labor right	[17,20]	Soil quality	[17,20]
Concentration of supplier	[18]	Decent live hood	[17,20]	Biodiversity	[17,20]
Concentration of slaughter	[18]	Traceability	[18,50]	Packaging	[18,51]
Product safety and quality	[20]	Animal well-being	[18,21]	The average of fuel needed to transport cattle from the farm to the slaughterhouses	[21,53]
Vulnerability	[20]	Equity	[20,56]	Energy from renewables	[52]
Productivity	[21]	Environmental service provider	[50,51]	Water deprivation	[48]
Diversity and structure industry	[21]	Productivity	[49,51]	Water resource	[19]
ROI	[51]	Mortality rate	[19]	Solid waste	[55]
Self-sufficiency	[21]	Frequency of conflicts related to the fattening of beef cattle	[16]	Cowshed cleanliness	[16]
Gross value added per workforce	[54]	Frequency of beef cattle extension and training	[16]	Agricultural land use change	[17]
Share of large enterprise	[54]	Alternative business	[16]	Insensitive agricultural/farming model	[18]
Consumption pattern and demand for poultry product	[56]	Time allocation uses for beef cattle flattening	[16]	Reverse logistics	[53]
Innovation	[51]	Social reputation	[18]	Eco production	[50]
Return on investment	[51]	Cultural diversity	[20]	Inventory cycle	[50]
Utilization order	[50]	Level of education of the employee	[21]	Inventory management	[50]
Traceability	[50]	Number of community-company partnership	[52]	Defective order	[50]
Market competitiveness	[49]	Average wage	[54]	Satisfaction level (backorder, delays)	[50]
Sustainability expenditure	[49]	Management level with a specific environmental responsibility	[53]	Environmental compliance	[49]
Customer price	[55]	Perfect order delivery	[53]	Supplier assessment	[49]
		Product life remaining	[53]	Photochemical	[55]
		Number of green products	[53]	Ozone creation potential	[55]
		Sustainability reports	[51]		
		Achieved objectives	[51]		
		Labor cost	[51]		
		Employee turnover rate	[51]		
		Rate defective product	[51]		
		Customer issue and complain	[51]		
		Ethical trading	[50]		
		Human rights and anticorruption	[49]		
		Social compliance	[49]		

The result in Table-1 indicated that the top five indicators when measuring economic conditions consist of profitability, income distribution, investment cost (such as material cost, energy cost, cost saved by the enterprises, operational and capital cost, insurance cost, and research and development cost), economic growth (local and or national growth), and rate per employee, giving some suggestions about quality, social and environmental issues, health, and safety. Then, the top seven indicators for measuring social condition consist of food safety, human health and safety, the number of employees owned by the enterprises, working conditions, gender equality, animal welfare, and the number of employees to be trained, whereas the top seven indicators for measuring environmental condition consist of energy used, global warming–GHG emissions, water consumption, land used, waste disposal, material used, and waste utilization. The previous authors are more in agreement on a type of indicator belonging to the environmental dimension than they are on that belonging to the economic and social dimensions. Indicators related to energy, water, and land used appear several times under different names. As an example, water quality, water deprivation, and water resources are used for water consumption. Soil quality and agricultural land use change are used for land use. Then, the list of indicators in Table-1, especially the indicators used by most previous authors, becomes the primary consideration in choosing the indicators used in this study. However, the ease of understanding of indicators by the farmers and the availability of primary and secondary data remain the main considerations in deciding the final indicators to be used. In detail, the indicators used for this study as well as their scale are shown in Table-2 [16-21,48-56] and Table-3.

**Table-2:** The indicators for measuring sustainability in beef supply chain.

Dimensions/indicators	Reference	Suggested quantification method (Yearly)
**Farm’s Level**		
Economic dimension		
Economic growth-added value per farm (PE1)	[17,19,20]	PE1=(The number of beef cattle×The average selling price of beef cattle)/(The number of farms)
Income distribution–percentage of the number of large–scale beef cattle farms compared to the total number of farms (PE2)	[16,19,49,50]	PE2=(The number of large–scale beef cattle farms)/(The number of farms)×100%
Self–sufficiency – the comparison between supply and demand of beef meat (PE3)	[21]	PE3=(The number of beef slaughterhouse capacity for a year×The average weight of beef cattle (Kg))/(The amount of beef consumption per capita (Kg)×Total population)×100%
Investment cost – the percentage of beef cattle covered by insurance by the farmer (PE4)	[20,21,50,51]	PE4=(The number of beef cattle covered by insurance)/(The population of beef cattle)×100%
Social dimension		
The number of employees in the enterprise – the ratio between the number of beef cattle farmer to the total of the farms (PS1)	[21,49,50,52,54]	PS1=(The number of beef cattle farmers)/(The number of the farms)
Gender equality – the comparison between female farmers to the total number of farmers (PS2)	[19,21,54,56]	PS2=(The number of female farmers)/(The number of the farmers)
Level of education of the employee – the percentage of farmers with level education higher than senior high school (PS3)	[21]	PS3=(The number of farmers with level education higher that senior high school)/(The number of the farmers)×100%
Animal welfare – percentage of beef cattle infected with the disease compared to the total number of beef cattle in one region (PS4)	[19,48,56]	PS4=(The number of beef cattle infected with the disease)/(The number of beef cattle)×100%
Environmental dimension		
Water consumption costs in using water for livestock activities (including cage cleanings) (PL1)	[21,50,52,54-56]	PL1=Water consumption costs per beef cattle per month ($)×The number of beef cattle×12
Energy used – cost in using electric for livestock activities (PL2)	[17,19,20,21,48-55]	PL2=Electric consumption per beef cattle per month (Kwh)×The number of beef cattle×Electricity cost per Kwh ($)
Global warming – the average of fuel needed to transport cattle from the farm to the slaughterhouses (PL3)	[21,53]	PL3=(The distance from the farm to slaughterhouse (km))/(The average of fuel needed per km (litter/km))×The price of fuel per litter ($)
**Beef Slaughterhouses’ Level**		
Economic dimension		
Economic growth – the added value earned per labor at beef slaughterhouses (RE1)	[17,19,20]	RE1=(((The average weight of the beef cattle x Selling price of the beef cattle per kg) – Selling price of the beef cattle)×The number of beef slaughterhouse capacity)/(The number of slaughterhouse labor)
Self-sufficiency – the comparison between supply and demand of beef meat (RE2) ****	[21]	RE2=(The number of beef slaughter capacity for a year×The average weight of beef cattle (Kg))/(The number of beef consumption per capita (Kg)×Total population)×100%
Social dimension		
The number of employees in the enterprise – the comparison between the number of employees worked at slaughterhouse compared to the total of slaughterhouse (RS1)	[21,49,50,51,54]	RS1=(The number of employee worked at slaughterhouse)/(The number of slaughterhouses)
Gender equality – the comparison between female worked at beef slaughterhouse to the total number of employees worked at beef slaughterhouse (RS2)	[19,21,54,56]	RS2=(The number of female employees)/(The number of the employees)
Level of education of the employee– the percentage of worker at beef slaughterhouse with level pf education higher than senior high school (RS3)	[21]	RS3=(The number of employees with level education higher that senior high school)/(The number of the employees)×100%
Animal welfare – percentage of beef cattle infected with the disease compared to the total number of beef cattle that go to the slaughterhouse (RS4)	[19,48,54]	RS4=(The number of beef cattle infected with the disease)/((The number of beef slaughter capacity for a year)×100%
Environmental dimension		
Water consumption costs in using water for beef slaughter activities (RL1)	[21,50,52,54-56]	RL1=Water consumption costs per month×12
Energy used – cost in using electric for beef slaughter activities (RL2)	[17,19,20,21,48-55]	RL2=Electric consumption per month (Kwh)×Electricity cost per Kwh ($)×12
Global warming – the average of fuel needed to transport cattle from the farm to the slaughterhouses (RL3)	[21,53]	PL3=(The distance from the farm to slaughterhouse (km))/(The average of fuel needed per km (liter/km))×The price of fuel per litter ($)

**Table 3:** Scale development for each indicator.

Dimensions/Indicators	1	2	3	4	5	6
Farms’ Level						
Economic dimension						
Economic growth – added value per farm (PE1) **	$ 1162.43-1929.74	$ 1929.75-2697.04	$ 2697.05-3464.34	$ 3464.35-4231.64	$ 4231.65-4998.95	USD $ 4998.96
Income distribution – percentage of the number of large–scale beef cattle farms compared to the total number of farms (PE2) ***	0%-7%	8%-15%	16%-23%	24%-31%	32%-39%	≥40%
Self–sufficiency – the comparison between supply and demand of beef meat (PE3) ****	0-19%	20%-39%	40%-59%	60%-79%	80%-99%	100%
Investment cost – the percentage of beef cattle covered by insurance by the farmer (PE4) ****	0-19%	20%-39%	40%-59%	60%-79%	80%-99%	100%
Social dimension						
The number of employees in the enterprise – the ratio between the number of beef cattle farmer to the total of the farms (PS1) **	0.51-0.95	0.96-1.40	1.41-1.86	1.87-2.31	2.32-2.76	≥2.77
Gender equality – the comparison between female farmers to the total number of farmers (PS2) ****	0-9%	10%-19%	20%-29%	30%-39%	40%-49%	50%
Level of education of the employee – the percentage of farmers with level education higher than senior high school (PS3) ****	0-19%	20%-39%	40%-59%	60%-79%	80%-99%	100%
Animal welfare-percentage of beef cattle infected with the disease compared to the total number of beef cattle in one region (PS4) ****	100%	80%-99%	60%-79%	40%-59%	20%-39%	0-19%
Environmental dimension						
Water consumption costs in using water for livestock activities (including cage cleanings) (PL1) *	≥$ 1,021,652.16	$ 853,009.86-1,021,652.15	$ 684,367.55-853,009.85	$ 515,725.24-684,367.54	$ 347,082.94-515,725.23	$ 178,440.63-347,082.93
Energy used – electricity usage costs for livestock activities (PL2) *	≥$ 5679.80	$ 4644.57-5679.79	$ 3609.34-4644.56	$ 2574.11 –3609.33	$ 1538.88-2574.10	$ 503.65-1538.87
Global warming – the average of fuel consumed to transport cattle from the farm to the slaughterhouses (PL3) *	≥$ 2157.22	$ 1811.88-2157.21	$ 1466.55-1811.87	$ 1122.58-1466.54	$ 773.96-1122.57	$ 345.34-773.95
Beef Slaughterhouses’ Level						
Economic dimension						
Economic growth – the added value earned per labor at beef slaughterhouses (RE1) **	$ 9533.17-14,299.80	$ 14,299.81-27,646.25	$ 27,646.26-40,992.71	$ 40,992.72-54,339.16	$ 54,339.17-67,685.62	≥$ 67,685.63
Self–sufficiency – the comparison between supply and demand of beef meat (RE2) ****	0-19%	20%-39%	40%-59%	60%-79%	80%-99%	100%
Social dimension						
The number of employees in the enterprise – the comparison between the number of employees worked at slaughterhouse compared to the total of slaughterhouse (RS1) **	0.50-3.7	3.8-7.0	7.1-10.3	10.4-13.6	13.7-16.9	≥17
Gender equality – the comparison between female worked at beef slaughterhouse to the total number of employees worked at beef slaughterhouse (RS2) ****	0-9%	10%-19%	20%-29%	30%-39%	40%-49%	0.5
Level of education of the employee– the percentage of worker at beef slaughterhouse with level of education higher than senior high school (RS3) ****	0-19%	20%-39%	40%-59%	60%-79%	80%-99%	100%
Animal welfare – percentage of beef cattle infected with the disease compared to the total number of beef cattle that go to the slaughterhouse (RS4) ****	100%	80%-99%	60%-79%	40%-59%	20%-39%	0-19%
Environmental dimension						
Water consumption costs in using water for beef slaughter activities (RL1) *	≥$ 414.43	$ 336.68-414.42	$ 258.94-336.67	$ 181.19-258.93	$ 103.45-181.18	$ 25.7-103.44
Energy used – electricity usage costs for beef slaughter activities (RL2) *	≥$ 420.16	$ 336.12-420.15	$ 252.09-336.12	$ 168.06-252.08	$ 84.03-168.05	$ 0-$ 84.02
Global warming – the average of fuel consumed to transport cattle from the farm to the slaughterhouses (RL3) *	≥$ 2157.22	$ 1811.88-2157.21	$ 1466.55-1811.87	$ 1122.58-1466.54	$ 773.96-1122.57	$ 345.34-773.95

* =Source of scale is primary data;

** =Source of scale is secondary data from Central Java Livestock Service, 2018;

*** =Source of scale is previous research from Yakovleva [54];

**** =Scale is developed by dividing the range from 0 to 100% or that from 0 to 50% into six scale portions

### RAPBEEF

RAPBEEF is a tool for rapid assessment of the sustainability status of beef supply chains using the Multidimensional Scaling (MDS). It is multivariate analysis technique that can help researchers interpret the relationship between several variables. One of the advantages of MDS is that this technique can model nonlinear relationships among variables, can handle nominal or ordinal data, and does not require multivariate normality [57]. Then, RAPBEEF is a modification of the Rapid Appraisal for Fisheries (RAPFISH), which has been under development at the Fisheries Centre at the University of British Columbia since 1998. RAPFISH is a statistical technique for rapid appraisal of the relative status of entities (=fisheries), judged quantitatively against predefined sets of attributes grouped into “evaluation fields” or disciplines. Approximate scores for each attribute are awarded on a scale from the worst to the best possible imaginable. Subsequent ordination is referenced to this scale, from “good,” or 100%, the best possible score, to “bad,” or 0%, the worst possible score [58].

The modification of the RAPFISH method for rapid appraisal of the relative status of entities other than fisheries can be found from several researchers [16,59-64]. Some of these researchers modify the name of RAPFISH according to the entity or subject of their research; the others only use the RAPFISH method without modifying the name. In this case, Syamsu *et al*. [59] modify the name of RAPFISH to RAP-Intrasadi (Rapid Appraisal Integration Beef Cattle and Paddy); Kapa and Suyadi [60] modify RAPFISH into Rap-UTSP-Laker (Rapid Appraisal for MDS); Sidabalok *et al*. [65] modify the RAPFISH method to Rap-slaughterhouse; and Arsyad *et al*. [64] modify the RAPFISH method into Rap-BANGKAPET (Rapid Appraisal Development of Animal Husbandry Development). Although these researchers used a similar method, and most of them used five dimensions (technological, economic, sociocultural, legal and institutional, and ecological) to assess the sustainability status of certain entities, sometimes, they did not use similar dimensions or even indicators. As an example, Parmawati [63] used the RAPFISH method to assess the sustainability of management and rural agropolitan development on the basis of five dimensions (human, natural, financial, physical, and social) and 25 indicators. Then, both Syamsu *et al*. [59] and Arsyad *et al*. [64] used the RAPFISH method to assess sustainability in the context of the beef industry. Syamsu *et al*. [59] assessed the sustainability status of the integration between beef cattle and paddy on the basis of four dimensions (technology, economic, sociocultural, and ecological) and 34 indicators. In contrast, Arsyad *et al*. [64] assessed the sustainability status on the integration between beef cattle and agricultural areas on the basis of five dimensions (technological, economic, sociocultural, legal and institutional, and ecological) and 53 indicators. It can be seen that RAPFISH is flexible enough to be used in different entities and different indicators. Based on this condition, different from Syamsu *et al*. [59] and Arsyad *et al*. [64], this research study prefers to use three components of sustainability from Elkington [37,38] (economic, social, and environmental) to assess the sustainability status on the beef supply chain, which consists of the beef cattle producer (the farmers) and the meat producer (slaughterhouse or butcher house) chain. Moreover, 11 and nine indicators are used to assess the sustainability status of farmers and slaughterhouse or butcher house chains, respectively.

### Object and sample of research

This research study used three regencies in Central Java Province to measure the sustainability of the beef supply chain (Semarang, Boyolali, and Sragen). These three regencies were selected on the basis of their capacity for beef meat production. Based on Central Java Livestock Services [66], Central Java is the third-highest beef meat producer in Indonesia after East Java and West Java. Then, the Semarang Regency, as the capital of Central Java Province, can produce 2,296,311 tons of beef meat from 49,172 heads of beef cattle. Boyolali Regency produces 209,230 tons of beef meat from 86,988 heads of beef cattle. Sragen Regency produces 1,423,045 tons of beef meat from 86,620 heads of beef cattle.

In this case, the number of beef farmers or beef slaughterhouses sampled to get primary data was not proportional to the total number of beef farmers or beef slaughterhouses located in each region as this research only takes a sample from those willing to participate. In the beginning, this research study applied nonprobability purposive samplings to select the beef cattle farmers and the beef slaughterhouse. Hence, this selection was based on specific characteristics. The farmers should have owned beef cattle and have been breeders for at least 2 years. Then, the slaughterhouse should be one with clear legality provided by the government in each regency. Based on this characteristic, the questionnaire for measuring the sustainability of the beef supply chain is addressed to 160 beef farmers and five beef slaughterhouses in each regency. We try to increase the response rate using several ways suggested by studies on survey research [67,68]. Each questionnaire was supplemented by a cover letter indicating the study purpose and potential contributions. The letter also assured complete confidentiality to the respondents. Follow-up calls were made to encourage completion and return of questionnaires and to clarify any questions that potentially had arisen [68]. After several reminders by phone calls and e-mails, we received 160 and five completed and usable questionnaires from beef farmers and beef slaughterhouses, respectively. In detail, the sample of this research comprises one beef slaughterhouse located at Boyolali, four located at Semarang, and one located at Sragen. Then, 30 beef farmers are located at Boyolali, 30 at Semarang, and 100 at Sragen. Based on the number of beef cattle owned by the farmers, among 160 beef farmers, 108 (67.5%) farmers have one to two head of beef cattle, 41 (25.63%) farmers have three to nine head of beef cattle, and 11 (6.88%) farmers have more than 10 head of beef cattle. The response rate ranged from 20% to 95%.

This research study also conducted nonprobability purposive sampling to choose respondents who filled out the questionnaire regarding the policies that should be implemented to improve beef supply chain sustainability (the questionnaire in the Delphi method). These respondents should have adequate information and pieces of knowledge concerning the situations of the beef supply chain. The respondents consisted of the head of the technical implementation unit for various livestock businesses and slaughterhouses, head of the livestock cultivation section, and the head of the animal breeding section.

### Indicators and measurement scale

Indicators for measuring the sustainability of the beef supply chain at the farmers and beef slaughterhouse levels numbered 11 and nine, respectively, accommodating literature review results; ease of understanding of indicators by the farmers and management representatives of the beef slaughterhouse; quantifiability or numerical measurability (although it is a qualitative indicator); and the availability of relevant data toward examining indicator condition. At the farmer’s level, the economic dimension consisted of four indicators, the social dimension consisted of four indicators, and the environmental dimension consisted of two indicators. In the chicken slaughterhouse chain, the economic dimension consisted of three indicators, the social dimension consisted of four indicators, and the environmental dimension consisted of three indicators. The proposed indicators that were utilized to examine the sustainability of the beef supply chain at both chains were established following some indicators developed by several researchers [16,17,19-21,48-56].

Each indicator in measuring the sustainability of farm and beef slaughterhouse chains applies a six-point Likert scale. Although a higher score indicates a better condition (1=the worst condition and 6=the best condition), the six-point Likert scale has a different meaning, depending on the condition asked for each indicator. For example, the meaning of values 1-6 for the question related to “economic growth-added value per farm per year (PE1)” can be explained as follows: 1=USD 1162.43-USD 1929.74; 2=USD 1162.43-USD 1929.74; 3=USD 1929.75-USD 2697.04; 4=USD 2697.05-USD 3464.34; 5=USD 3464.35-USD 4231.64; 6=more than USD 4998.96. The indicators, their scale, and the equation to calculate each indicator for farms and beef slaughterhouses are listed in detail in Tables-2 and 3, respectively. Then, the source of scale development for each indicator is varied. The scales of indicators PL1, PL2, and PL3 are obtained from statistical processing of 160 farmers’ primary data, whereas the scale of RL1, RL2, and RL3 are obtained from statistical processing of five beef slaughterhouses’ primary data. The scale of PE1, PS1, RE1, and RS1 is developed under secondary data from Central Java Livestock Service [66]. The scale of indicator PE2 is developed from a previous study by Yakovleva [54]. Moreover, the scale of indicators PE3, PE4, PS2, PS3, PS4, RE2, RS2, RS3, and RS4 is developed by dividing the range from 0 to 100% or that from 0 to 50% into six scale portions.

### Data collection procedure

This research study used two sources of data, primary data and secondary data. The sources of primary data were the questionnaire and the results of the interview with selected beef cattle farmers, selected representative management of the beef slaughterhouse, and selected representatives of the Regional Department of Animal Husbandry and Fisheries. Then, secondary data were collected from published reports and information obtained from institutions involved in the beef supply chain, the Central Bureau of Statistics, and the Regional Department of Animal Husbandry and Fisheries. Secondary data, among others, were used to find the number of beef cattle, the selling price of beef cattle, the number of farms, the number of large-scale beef farmers, etc. (Table-3, for the source of the scale of each indicator).

This research study used two types of questionnaires to collect data. The first questionnaire is RAPBEEF score sheets, which was developed to assess the condition of three indicators of environmental dimensions at each level (beef farmers and slaughterhouse level). Three hundred and thirty copies of the first type of questionnaire were delivered to the selected beef farmers and representative management of the beef slaughterhouses. They were asked to describe the level of water used, electricity used, and fuel needed in 1 year. Personal interviews were conducted to accompany the distribution of the first questionnaire to identify further the actual value or the reason for which each respondent selects a specific scale in each indicator.

The second questionnaire is the Delphi questionnaire, which is a combination of semi-structured and closed-ended questionnaires. It is used to find consensus among a group of experts [69]. In this research study, the Delphi questionnaire is used to find consensus related to several policies to improve the sustainability of the beef supply chain. The distribution of the Delphi questionnaire took several rounds. In the first round, semi-structured questions are delivered to the expert to identify some proposed policies toward improving the sustainability of the beef supply chain. On the basis of information collected from the first round, the closed-ended questions are delivered for the second and subsequent rounds. This questionnaire intends to quantify the proposed policy’s priority level on the basis of earlier findings, usually by rating it with a five-point Likert scale (1=not a priority to 5=essential). Three copies of the second type of questionnaire were delivered to the Regional Department of Animal Husbandry and Fisheries representative.

### Data processing technique

This research study used RAPBEEF to assess the sustainability status of beef supply chains and the Delphi method to formulate the proposed policy to improve sustainability status. The RAPBEEF approach is carried out through several steps [58]. First, determine the indicators of each sustainability dimension and its scale through a literature review. Second, evaluate the condition of each indicator according to an ordinal scale (scoring). This evaluation is done on the basis of actual data conditions in the field, whether it be from the questionnaire, interview, or observation (primary data), or secondary data. Third, analyze ordination with MDS for sustainability evaluation in each dimension of the sustainability index scale. The sustainability index value was separated into four levels: The 0-25 range was within the unsustainable status, the 26-50 range was fairly unsustainable, the 51-75 range was moderately sustainable, and the 76-100 range was within good sustainability. Fourth, determine the goodness-of-fit of the model. It is done by observing the S-Stress value (by calculating the S and R2 values). A lower S value indicates high goodness of fit. The S value should be <0.25, and the R2 value should be close to 1.00. Fifth, calculate the Root Mean Square (RMS) as the leverage analysis to find sensitive indicators. The higher the value of RMS the greater the indicator effect on the level of sustainability. Sixth, and last, identify the random error of all dimensions through Monte Carlo analysis. The results of the Monte Carlo analysis were compared with those of MDS analysis. In this case, for a 95% confidence interval, the MDS result was sufficient if the differences between Monte Carlo and MDS analysis were <5%. Moreover, if the differences between the results from MDS and those from Monte Carlo analysis were relatively small (<1.00), the level of error in the analysis would be tolerable.

Then, this research study used the Delphi method to formulate the proposed policy to increase the sustainability status on the basis of consensus on two conditions. First, there is the consensus on the cause of sensitive indicators, and second, there is the consensus on the recommendation policy to overcome the cause of sensitive indicators. Several rounds were carried out by the Delphi method, and this round would be stopped if the consensus is reached. In this case, the level of consensus between the experts is measured by calculating Kendall’s coefficient of concordance (Kendall’s W). This coefficient ranges from 0 to 1. Strong consensus, indicated by a Kendall’s W value of more than 0.7; moderate consensus indicated by a Kendall’s W value equaling 0.5; weak consensus indicated by a Kendall’s W value of <0.3 [69,70].

## Results and Discussion

### The result of measuring the sustainability status of beef supply chain

Table-4 indicates the performance evaluation of each indicator according to secondary and primary data obtained from 160 beef cattle farmers and five representatives of the management of beef slaughterhouses. These tables describe the actual value of each indicator and the value of each indicator according to its scale.

**Table 4:** The result of performance evaluation of each indicator.

	Dimensions/indicators	The actual value			The value according to scale		
	
		Semarang	Boyolali	Sragen	Semarang	Boyolali	Sragen
Farm’s level	Economic dimension						
	Economic growth (PE1)	$ 143,09	$ 1.657,29	$ 2.174,84	1	1	1
	Income distribution (PE2)	2.43%	2.35%	0.93%	1	1	1
	Self-sufficiency (PE3)	170,28%	519,11%	88,70%	6	6	5
	Investment cost (PE4)	1.67%	0.96%	0.17%	1	1	1
	Social dimension						
	The number of employees in the enterprise (PS1)	1094	1039	1436	2	2	2
	Gender equality (PS2)	53.12%	46.72%	48.62%	3	3	3
	Level of education of the employee (PS3)	19.13%	24.89%	25.00%	3	3	3
	Animal welfare (PS4)	6.07%	1.41%	1.57%	6	6	6
	Environmental dimension						
	Water consumption (PL1)	$ 520.662,86	$ 925.988,57	$ 436.699,21	5	2	5
	Energy used (PL2)	$ 1.668,25	$ 3.157,93	$ 4.877,75	5	4	2
	Global warming (PL3)	$ 846,30	$ 1.913,94	$ 1.300,83	5	2	4
Beef slaughterhouses level	Economic dimension						
	Economic growth (RE1)	$ 3.215,13	$ 23.342,99	$ 12.288,95	1	2	1
	Self-sufficiency (RE2)	170.28%	519.11%	88.70%	6	6	5
	Social dimension						
	The number of employees in the enterprise compared to the total of slaughterhouse (RS1)	10.75	17	5	4	6	2
	Gender equality (RS2)	27.91%	23.53%	100.00%	2	2	6
	Level of education of the employee (RS3)	4.65%	0.00%	0.00%	1	1	1
	Animal welfare (RS4)	1.94%	0.13%	7.88%	1	1	1
	Environmental dimension						
	Water consumption (RL1)	$ 367.86	$ 142.86	$ 180.00	2	5	5
	Energy used (RL2)	$ 362.86	$ 142.86	$ 100.29	2	5	5
	Global warming (RL3)	$ 846.30	$ 1.913,94	$ 1.300,83	5	2	4

**Figure-2:**
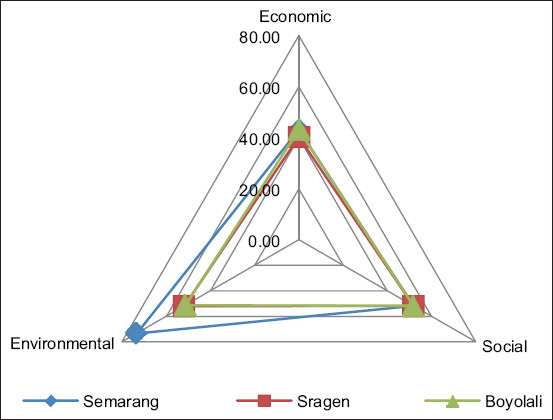
Kite Diagram of three dimensions of sustainability of beef cattle farms at Semarang, Sragen, and Boyolali.

**Figure-3:**
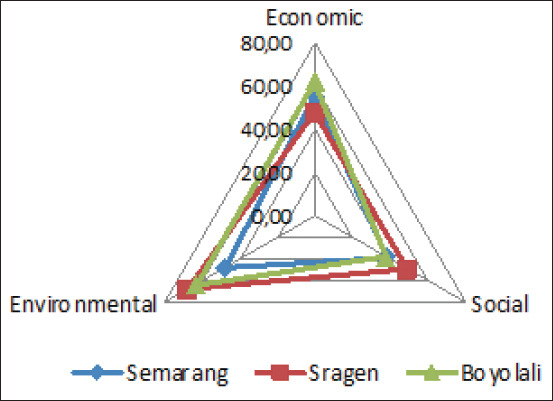
Kite Diagram of Three Dimensions of Sustainability of Beef Slaughterhouse at Semarang, Sragen, and Boyolali.

**Figure-4:**
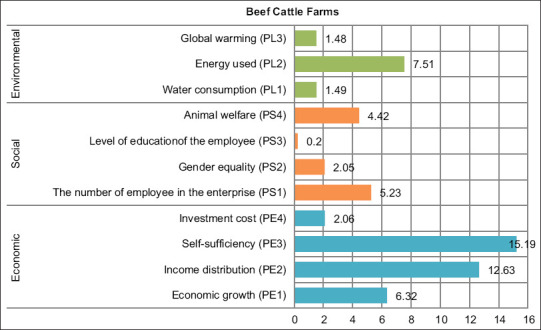
Indicator leverage analysis for sustainability status in beef cattle farms level.

**Figure-5:**
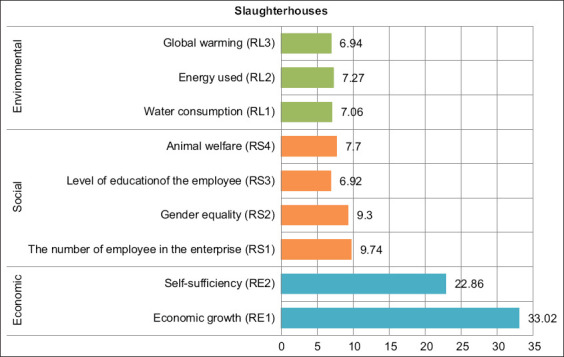
Indicator leverage analysis for sustainability status at beef slaughterhouse.

Then, based on the performance evaluation of each indicator, the result of analysis of ordination with MDS (sustainability index of each dimension), and the S-Stress and R2 values, as well as the result of Monte Carlo analysis are shown in Tables-5 and 6 as well as in Figures-2 and 3. Then, the value of RMS is calculated to identify the sensitive indicators as shown in Figures-4 and 5.

**Table 5:** MDS result, Monte Carlo, S-Stress, and R2 at beef cattle farms.

Dimension	Sustainability index of beef cattle farm										

	Semarang			Sragen			Boyolali			S-stress	R2
		
	MDS	Monte Carlo	Diff	MDS	Monte Carlo	Diff	MDS	Monte Carlo	Diff		
Multidimension	56.14***	56.13	0.01	48.02**	47.75	0.28	48.77**	48.50	0.27		
Economics	43.18**	42.63	0.55	40.27**	40.31	0.04	43.18**	42.80	0.38	0.17	0.92
Social	51.82***	51.34	0.48	51.82***	51.49	0.33	51.82***	51.81	0.01	0.21	0.92
Environmental	73.42***	74.41	0.99	51.98***	51.44	0.54	51.3***	50.89	0.41	0.23	0.88

*=Unsustainable status;

** =Fairly unsustainable;

*** =Moderately sustainable;

****=Good sustainable

**Table 6:** MDS Result, Monte Carlo, S-Stress, and R^2^ at Beef Slaughterhouse.

Dimension	Sustainability index of slaughterhouse										

	Semarang			Sragen			Boyolali			Statistics	R2
		
	MDS	Monte Carlo	Diff	MDS	Monte Carlo	Diff	MDS	Monte Carlo	Diff		
Multidimension	47.05**	46.57	0.48	54.83***	54.00	0.83	54.19***	53.47	0.72		
Economics	55.17***	53.86	1.31	47.65**	46.52	1.13	61.69***	59.63	2.06	0.21	0.95
Social	38.22**	38.38	0.16	49.00**	47.60	1.40	37.56**	38.04	0.48	0.19	0.92
Environmental	47.75**	47.47	0.28	67.85***	67.88	0.03	63.32***	62.75	0.57	0.23	0.92

*=Unsustainable status;

** =Fairly unsustainable;

*** =Moderately sustainable;

****=Good sustainable

Table-5 indicates that the regional sustainability index value for multidimensional approaches was 56.14, 48.02, and 48.77 for beef cattle farms in Semarang, Sragen, and Boyolali, respectively. The value fell on a moderately sustainable level for beef cattle farms in Semarang and a fairly unsustainable level for beef cattle farms in Sragen and Boyolali. In line with the regional sustainability index value for the multidimensional approach, the sustainability index value for economic, social, and environmental dimensions for all regions has ranged between 40.27 and 73.42 and was falling on fairly unsustainable and moderately sustainable levels. Table-5 also indicates that the sustainability index value for economic, social, and environmental dimensions for the Semarang region tended to be higher than Sragen or Boyolali. Moreover, the results of the Monte Carlo calculation of the three dimensions show a slight difference value (0.01-0.99%) with the calculation of RAPBEEF. This situation shows that the results of analyses performed on the ordination of three-dimensional measurement for sustainability at beef cattle farms are stable enough. The result for S-Stress is <25%, and R2≈1, which means that the RAPBEEF calculation results for sustainability at the beef cattle farm can be accepted. Then, Kite Diagram in Figure-2 indicates that sustainable beef cattle farm effort in the Semarang region refers to the environmental dimension, which is the dimension with the highest analytical results (73,42), followed by the social and economic dimensions at 51.82 and 43.18, respectively, by value. In Sragen, the sustainable beef cattle farm effort refers to the environmental dimension, and in Boyolali, it refers to the social dimension, which is the dimension with the highest analytical result.

Table-6 indicates that the regional sustainability index values for multidimensional approaches were 47.05, 54.83, and 54.19 for beef slaughterhouses in Semarang, Sragen, and Boyolali, respectively. The value fell on fairly unsustainable levels for a beef slaughterhouse in Semarang and moderately sustainable levels for a beef slaughterhouse each in Sragen and Boyolali. Then, the sustainability index value for economic, social, and environmental dimensions for a beef slaughterhouse in three regions have ranged between 37.56 and 67.85. According to this value, part of the sustainability index value for the economic, social, and environmental dimensions of a beef slaughterhouse each in Semarang, Sragen, and Boyolali fell on fairly unsustainable levels, and the other fell on moderately sustainable levels. It seems, in general, that the sustainability of a beef cattle farm is better than that of a beef slaughterhouse. Then, the results of the Monte Carlo calculation of the three dimensions show a slight difference value (0.03-2.06%) with the calculation of RAPBEEF. This situation shows that the results of analyses performed on the ordination of three-dimensional measurement of sustainability at beef cattle farms are stable enough. The result for S-Stress is <25%, and R2≈1, meaning that the RAPBEEF calculation results for the sustainability of beef slaughterhouses can be accepted. Then, the Kite Diagram in Figure-3 indicates that sustainable beef slaughterhouse efforts in the Semarang region refers to the economic dimension, which is the dimension with the highest analytical results (55.17), followed by the environmental and social dimensions at 47.75 and 38.22, respectively, by value. In Sragen and Boyolali, sustainable beef slaughterhouse efforts refer to the environmental dimension, which is the dimension with the highest analytical results.

In the leverage analysis of beef cattle farms (Figure-4), RMS values indicated that the most sensitive indicator affecting the economic dimension was the comparison between supply and demand of beef meat (PE3, RMS=15.19), followed by the percentage of the number of large-scale beef cattle farms when compared with the total number of farms (PE2, RMS=12.63), then by added value per farm (PE1, RMS=6.32), and then by the percentage of beef cattle covered by insurance by the farmers (PE4; RMS=2.06). Then, the most sensitive indicator affecting the social dimension was the ratio between the number of beef cattle farmers to the total number of farms (PS1, RMS=5.23), followed by the percentage of beef cattle infected with disease when compared with the total number of beef cattle in one region (PS4, RMS=4.42), then by the comparison between female farmers to the total number of farmers (PS2, RMS=2.05), and then by the percentage of farmers with levels of education higher than senior high school (PS 3, RMS=0.20). The most sensitive indicator affecting the environment was electricity usage costs for livestock activities (PL2; RMS=7.51), followed by costs in using water for livestock activities (including cage cleanings) (PL1; RMS=1.49) and then by the average of fuel consumed to transport cattle from the farm to the slaughterhouses (PL3; RMS=1.48).

In the leverage analysis for beef slaughterhouses (Figure-5), RMS values indicate that the indicator “added value earned per labor at the beef slaughterhouse” (RE1; RMS=33.02) was the most sensitive indicator affecting the economic dimension when compared with the comparison between supply and demand of beef meat (RE2; RMS=22.86). Then, the most sensitive indicator affecting the social dimension was the comparison between the number of employees who worked at a slaughterhouse and the total number of workers at slaughterhouses (RS1; RMS=9.74), followed by the comparison between female workers at the beef slaughterhouse and the total number of employees working at the beef slaughterhouse (RS2; RMS=9.30), then by the percentage of beef cattle infected with disease when compared with the total number of beef cattle that go to the slaughterhouse (RS 4; RMS=7.70), and then by the percentage of workers at the beef slaughterhouse with a level of education higher than senior high school (RS3; RMS=6.92). The most sensitive indicator affecting the environmental dimension was electricity usage costs for beef slaughter activities (RL2; RMS=7.27), followed by costs in using water for beef slaughter activities (RL1; RMS=7.06) and then by the average of fuel consumed to transport cattle from the farm to the slaughterhouses (RL3; RMS=6.94).

### Policy recommendation based on the Delphi method

The findings obtained from the leverage analysis, which indicates the most sensitive indicator affecting the sustainability of each dimension, were discussed with some experts to find alternative policies that could increase the sustainability level of those indicators (PE3, PS1, and PL2 for beef cattle farms; RE1, RS1, and RL2 for beef slaughterhouse). The Delphi method was used to formulate the policies for those indicators. In this research study, the Delphi study was administered in two rounds. In the first round, semi-structured questions were distributed to the experts to identify some proposed policies related to the most sensitive indicator affecting the sustainability of each dimension. Briefly, several proposed policies resulted from the first round of beef cattle farms can be explained as follows.

a.The experts proposed two policies in relation to self-sufficiency (the difference between the demand for beef cattle and the total population of beef cattle). First, governments should focus on a program enhancing beef cattle production through two mating systems, AI and natural mating; both can maximize the ability of beef cattle to produce calves (POP 1). Second, facilitate the building of a cattle beef community husbandry center (SPR), which can consolidate small breeders to become a collective business managed in one management to facilitate their services (e.g., animal reproduction and health facilities, technical training and assistance, and business partnerships with private companies); hence, the farmers can produce more output per productive animal unit on the farm and more output per productive acre on the farm (POP 2).
b.In relation to the number of employees in the enterprise (the ratio between the number of beef cattle farmers to the total of the farms), the experts proposed two policies. First, the government should focus on retaining as well as increasing the number of beef cattle farmers by providing subsidized credit to support farmers increase their scale of production as well as support training/assistances/technology in keeping cattle so that the farmers can produce more output per productive animal unit on the farm and more output per productive acre on the farm (POP 3). Second, the government needs to establish policies to protect beef cattle farmers from middlemen (improve the farmer’s bargaining position). The farmers can get more benefits and make the livestock business more attractive (POP 4).
c.In relation to energy used (electricity usage costs for livestock activities), the experts proposed two policies. First, educate beef farmers on reducing electricity consumption on livestock activities (POP 5). Second, facilitate large-scale beef cattle farmers to build biogas from livestock waste to substitute for electrical energy (POP 6).

Several proposed policies resulted from the first round of beef slaughterhouses can be explained as follows.

a.In relation to economic growth (the added value earned per labor at beef slaughterhouses), the experts proposed four policies. First, the slaughterhouse should focus on getting a veterinary control number (NKV) certificate published by the local farming office as a prerequisite toward reaching a higher market demand because increasing added value is related to the total output produced by the enterprise. The government will gradually apply NKV ownership to the distribution of food products by the producer (Regulation of Ministry of Agriculture Republic of Indonesia No. 11/2020) (POR 1). Second, ensure that the slaughterhouse has facilities following the Regulation of the Minister of Agriculture Republic of Indonesia No. 14/2010 concerning the requirements for ruminant animal cutting houses and meat-cutting plants to get the best quality of beef meat (POR 2). Third, facilitate the building of an integrated livestock area consisting of a livestock center, animal market, and slaughterhouse to increase users of slaughterhouse services (POR 3). Fourth, increase supervision to prevent the slaughter of cattle outside legal slaughterhouses (increase the number of legally slaughtered cattle as well as the output produced by legal slaughtering) (POR 4).
b.The experts proposed one policy in relation to the number of employees in the enterprise (comparing the number of employees who worked at a slaughterhouse with the total number of slaughterhouse workers). To retain excellent workers at beef slaughterhouses, as well as attract new workers, the government needs to review the condition of the slaughterhouses as the workers are very close to several hazards including exposure to high noise levels, dangerous equipment, slippery floors, musculoskeletal disorders, and hazardous chemicals (including ammonia) (POR 5).
c.In relation to energy used (electricity usage costs for beef slaughter activities), the experts proposed two policies. First, educate managements of beef slaughterhouses on the strategies to reduce electricity consumption on livestock activities (POR 6). Second, the government needs to create a regulation that requires the management of beef slaughterhouses to utilize beef cattle waste as biogas to substitute electrical energy (POR 7).

Then, based on the proposed policies gathered from the first round, closed-ended questions were used for the second round. The results for the second round are summarized in Table-7.

**Table 7:** Second round of the Delphi method.

Expert/Policy	Beef cattle farms						Beef slaughterhouse						
	
	POP 1	POP2	POP 3	POP 4	POP 5	POP 6	POR1	POR 2	POR3	POR4	POR5	POR6	POR7
E1	5	3	4	4	4	5	5	5	5	5	5	4	5
E2	5	4	3	5	4	4	5	5	5	5	5	4	4
E3	5	4	3	5	4	5	5	4	5	5	5	4	5
Mean	5.0	3.7	3.3	4.7	4.0	4.7	5.0	4.7	5.0	5.0	5.0	4.0	4.7

n=9; Kendall’s W (Kendall’s Coefficient of Concordance)=0.795; Chi-square=33.400; df 14; Asymp. Sig.=0.003

Table-7 shows the result of Kendall’s coefficient of concordance (Kendall’s W) test for the second round. The Delphi method does not need to be used further in the third round because Kendall’s W in the second round is 0.795 or more than 0.5. Kendall’s W denotes the consensus level among the participants [71], and it ranges from 0 to 1, indicating the degree of consensus reached by the panel. A Kendall’s W of more than 0.7 shows strong consensus; a Kendall’s W of 0.5 shows moderate consensus; and a Kendall’s W of <0.3 shows weak consensus [71]. A review of the data from a second round shows that all alternative policies could be regarded as an essential alternative policy because none of those policies have an average value of <3. Hence, the final alternative policies according to their rank are as follows: Governments should focus on programs enhancing beef cattle production through two mating systems (POP 1); the slaughterhouse should focus on getting a veterinary control number (NKV) certificate published by the local farming office (POR 1); facilitate the building of an integrated livestock area (POR 3); increase supervision to prevent the slaughter of cattle outside legal slaughterhouses (POR 4); the government needs to review the condition of the slaughterhouses as the workers work very closely with several hazards (POR 5); government needs to establish policies to protect beef cattle farmers from middlemen (POP 4); facilitate the production of biogas from livestock waste generated by large-scale beef cattle farms (POP 6); ensure that the slaughterhouse has facilities in accordance with the Regulation of the Minister of Agriculture Republic of Indonesia No. 14/2010 (POR 2); the government needs to create a regulation that requires the management of beef slaughterhouse to utilize beef cattle waste as biogas (POR 7); educate beef farmers and representative managements of slaughterhouse strategies to reduce electricity consumption on livestock activities (POP 5) (POR 6); facilitate the building of a cattle beef community husbandry center (SPR) (POP 2); and government should focus on retaining as well as increasing the number of beef cattle farmers by providing subsidized credit (POP 3).

## Conclusion

This research study intended to find suitable indicators and their scale for measuring the level of sustainability of the beef supply chain. It also tried to apply RAPBEEF as a measurement system in a specific beef supply chain and formulate recommendations to increase the sustainability of the beef supply chain. Based on a literature review from 15 previous researchers, this study identified 11 and nine indicators, respectively, belonging to economic, social, and environmental dimensions to measure sustainability performance at beef farms and slaughterhouses. Measurement results revealed that the achievement of beef supply chain sustainability requires targeted efforts with the deployment of several policies because the current status of sustainability in beef farms and beef slaughterhouses only fell on moderately sustainable and fairly unsustainable levels. Although all the surveyed regions in this study can meet the regional needs of beef meat on their own or could even distribute the excess to other regions, none of the beef supply chains of the surveyed region fell on good sustainability. In detail, at the beef farm level, the regional sustainability index value fell on moderately sustainable levels in Semarang and fairly unsustainable levels in Sragen and Boyolali. Then, at the beef slaughterhouse, the regional sustainability index value fell on fairly unsustainable levels in Semarang and moderately sustainable levels in Sragen and Boyolali. Semarang has more sustainability in beef cattle farms, whereas Sragen and Boyolali have more sustainability in the beef slaughterhouse. Moreover, the sustainability of beef farms and slaughterhouse levels at three surveyed regions was due to “fairly unsustainable” on one or two dimensions of economic, social, and environmental indicators. Efforts to increase sustainability status levels focused on the sensitive indicators (the indicators that have a more significant effect on the sustainability of each dimension), such as self-sufficiency (the difference between the demand for beef cattle and the total population of beef cattle), the number of employees in the enterprise (the ratio between the number of beef cattle farmers to the total of the farms), energy used (electricity usage costs for livestock activities or beef slaughter activities), economic growth (the added value earned per labor unit at beef slaughterhouses), and the number of employees in the enterprise (the comparison between the number of employees who worked at a slaughterhouse and the total number of workers at slaughterhouses).

This research study has some implications for increasing the sustainability of the beef supply chain because beef production has not kept pace with demand growth, and cattle farming in Indonesia is categorized as unsustainable [72]. Since sustainability is the achievement of economic, environmental, and social goals simultaneously, represented by various performance indicators, the government or policymakers should focus on sensitive indicators resulting from this research. Based on that situation, the government should maximize the conditions addressed to increase the domestic cattle population and beef meat production and improve the economic condition of smallholder farmers. Because the ratio between the number of beef cattle farmers belongs to the sensitive indicator of protecting and retaining the number of farmers, the government regulation should improve the bargaining position of the farmers by covering activities from upstream (especially reproduction and feeding management, capital, small schemes of credit, and institutions) to downstream (products and marketing). From a theoretical point of view, this research study has a significant theoretical implication on the sustainability literature. The primary contribution of this research is the development of a robust framework to measure sustainability performance in the beef supply chain by adapting the RAPFISH method with a new indicator, a new scale, and a new object. The proposed framework can help researchers derive new ideas to adopt the RAPFISH method for other research objects and refine, extend, and enhance this framework. This framework offers an excellent opportunity for scholars to conduct tests in other beef supply chains.

### Limitations

This study has several limitations. First, limited beef cattle farmers and slaughterhouses were included in the study sample to develop the scale of specific indicators (PL1, PL2, PL3, RL1, RL2, and RL3) and to measure those indicators’ conditions. Only 160 beef cattle farmers and five beef slaughterhouses located in Semarang, Sragen, and Boyolali were considered. The limited sample of this research study can cause bias in our scale and the results of measurement because of the condition of the surveyed beef cattle farmers and beef slaughterhouse. Second, the beef supply chain is the object of the study, which focuses only at the farm and beef slaughterhouse levels. This study excludes consumers (such as restaurants, hotels, catering and food industries, wet markets, supermarkets, hypermarkets, etc.) as part of measuring the sustainability level of the beef supply chain. Given this limitation, future research should enhance the sample size of beef cattle farmers and slaughterhouses located in Semarang, Sragen, and Boyolali, as well as beef cattle farmers and slaughterhouses located in other regions of Central Java Province and even those from other provinces. Future research should also compare the level of sustainability status between regions because the sustainability of beef cattle farms can be recognized through a regional approach by considering the region’s potential [72]. Moreover, future studies should enhance the measurement of sustainability of the beef supply chain by including other factors related to consumers and other participating actors, such as governments.

## Authors’ Contributions

AS: Designed and managed the study and wrote the manuscript. RP and ANA: Collected and analyzed the samples. HS and BT: Reviewed and corrected the manuscript. All authors have read and approved the final manuscript.
